# Evaluation of Dietary Bioactive Agents Against Aflatoxin B1 and Ochratoxin A-Induced Duodenal Toxicity in Rats

**DOI:** 10.3390/foods14101793

**Published:** 2025-05-18

**Authors:** Sarra Rafai, Alessandra Cimbalo, Lara Manyes

**Affiliations:** Biotech Agrifood, Faculty of Pharmacy and Food Sciences, Universitat de València, Avda, Vicent Andrés Estellés s/n, 46100 Burjassot, Spain; sarra.rafai@uv.es (S.R.); lara.manyes@uv.es (L.M.)

**Keywords:** bioactive compound, mycotoxin, gene expression, apoptosis, biomarker

## Abstract

Aflatoxin B1 (AFB1) and Ochratoxin A (OTA) are two of the most potent mycotoxins, recognized for their severe toxicity. In recent years, the consumption of bioactive substances has proven to be a valuable ally in combating their harmful effects on human health. For this purpose, this study evaluates the protective effects of fermented whey (FW) and pumpkin (P), as functional ingredients in bread, on duodenum tissue against sub-chronic toxicity induced by AFB1 and OTA. Nine groups of male and female Wistar rats (*n* = 5 per sex/group) were exposed to different combinations of AFB1, OTA, FW, and P for 28 days. The gene expression of apoptotic and antioxidant markers, including p53, Bax, Hmox1, NF-κB, and occludin, was measured by quantitative real-time PCR (RT-qPCR). AFB1 + OTA exposure led to an increased expression of p53 and NF-κB, with the downregulation of Bax and Hmox1. Occludin expression, which supports tight junction integrity, remained largely unaffected. Supplementation with FW and FW + P modulated gene expression favorably, offering protection against AFB1 and OTA toxicity. These bioactive components effectively mitigated oxidative stress and apoptosis in duodenal tissue. Notably, the results indicate that the protective effects of FW and P are not sex-dependent. These findings highlight the potential of FW and P as functional ingredients in combating the toxic effects of AFB1 and OTA in vivo.

## 1. Introduction

Mycotoxins are one of the most toxic compounds and present a global concern. They are secondary metabolites produced by various species of filamentous fungi that contaminate staple food, whether in their raw state or food products. Due to their high resistance to food processing approaches, they inevitably enter the food chain either directly by eating contaminated food or indirectly from animals that are fed contaminated feed. For the past decades, studies have identified several hundred various mycotoxins. However, aflatoxin B1 (AFB1) and ochratoxin A (OTA) are some of the most prominent mycotoxins in their toxin families. They present a serious concern to human and animal health as they have been proved to cause acute reactions, such as symptoms of severe illness appearing quickly, or chronic effects linked to long term diseases, such as cancers and immune deficiency [1].

AFB1 is a secondary metabolite produced by certain fungi, particularly *Aspergillus flavus* and *A. parasiticus* [2]. Being the most potent of the five aflatoxins, it constitutes a serious public health concern due to its high toxicity and widespread occurrence in food. Classified as a Group 1 carcinogen by the IARC, acute exposure has been associated with severe clinical manifestations, including gastrointestinal distress, neurological symptoms, pulmonary and cerebral edema, and in extreme cases, coma or death [3].

Chronic low-dose exposure to AFB1 is also concerning, as it can lead to liver cancer, cirrhosis, and hepatitis, and may affect other organs such as the kidneys, pancreas, bladder, bones, and viscera. It has also been associated with congenital abnormalities and immunosuppression [3,4,5]. AFB1 is primarily metabolized in the liver by CYP450 enzymes into exo-8,9-epoxide (AFBO), a highly reactive compound responsible for its genotoxicity. AFBO binds strongly to DNA, causing mutations—especially at the third base of codon 249 in the p53 tumor suppressor gene involved in apoptosis [6]. Moreover, AFBO interacts with other cellular macromolecules, including proteins and phospholipids, causing various metabolic and structural disturbances [7].

OTA is also a mycotoxin produced by the fungi of the *Aspergillus* and *Penicillium* genera [8]. It can contaminate various food items such as cereals, nuts, meat, coffee, and wine. It is the most toxic ochratoxin and is classified as a Group 2B carcinogen by IARC. OTA has shown nephrotoxic, hepatotoxic, teratogenic, and immunomodulatory effects in animals, with studies in rats linking it to nephropathy [9,10]. It has also been associated with adverse effects on the liver, immune system, and nervous system [11]. OTA is primarily absorbed in the proximal jejunum and distributed to organs such as the liver and kidneys [12]. Its toxicity involves increased NADPH and P450 activity, caspase pathway activation, and ROS production leading to apoptosis [13]. Additionally, OTA inhibits protein synthesis by blocking phenylalanine tRNA synthetase due to its structural similarity to phenylalanine [14].

Completely eliminating mycotoxins from the diet remains a major challenge [15]. To counter their harmful effects, the inclusion of bioactive ingredients is increasingly recommended [16]. Among them, fermented whey (FW) and pumpkin have shown promising protective effects [17]. FW, enhanced through lactic acid bacteria fermentation, contains peptides, phenolic acids, and bacteriocins, offering antioxidant, antifungal, and antimicrobial properties [18]. It has been shown to reduce the intestinal bioaccessibility of AFB1 and OTA and to modulate their toxicity in both in vitro and in vivo models [19,20].

Pumpkin is also rich in health-promoting compounds such as carotenoids, polyphenols, organic acids, and essential vitamins [21]. A transcriptomic study demonstrated the anti-inflammatory and protective effects of the FW and pumpkin in combination against AFB1 and OTA toxicity, highlighting its potential for food industry applications [22].

The main goal of this study is to evaluate the preventive effects of FW and pumpkin in the duodenum of rats exposed to AFB1 and OTA-contaminated feed over 28 days, individually and in combination, through gene expression analysis using real-time quantitative PCR. The study focuses on the expression of key genes involved in apoptosis, including the tumor suppressor p53, pro-apoptotic Bax, anti-apoptotic and anti-inflammatory NF-κB, oxidative stress marker Hmox1, and occludin, a critical gene for maintaining intestinal barrier integrity.

## 2. Materials and Methods

### 2.1. Reagents and Equipment

The reagents used, methanol and isopropanol, were supplied by Fisher Scientific (Madrid, Spain). For RNA extraction, the TRIzol^TM^ reagent was purchased from Invitrogen^TM^ (Carlsbad, CA, USA), while the ReliaPrep^TM^ RNA Miniprep System kit from Promega (Madison, WI, USA) was used for purification. Complementary DNA (cDNA) was obtained using the TaqMan Reverse Transcription Reagents kit (Thermo Fisher Scientific, Waltham, MA, USA). PowerUp^TM^ SYBR^TM^ Green for qPCR analysis was purchased from Applied Biosystems (Carlsbad, CA, USA).

### 2.2. In Vivo Study and Experimental Protocol

A total of 90 Wistar rats (45 males and 45 females, weighing between 260 and 340 g) were acquired from Pharmacy animal facility (Universitat de València, Spain), and the institution Animal Care and Use Committee of the University of Valencia approved all animal procedures (protocol no. A13338818442265). The study was conducted in a room with controlled environmental conditions, with a temperature of 22 °C and relative humidity between 45% and 65%, which were suitable for the species. To maintain sterile conditions, nitrile gloves and FFP3 masks were worn during all procedures, including the handling of animals and contaminated samples.

Subsequently, the rats were randomly divided into 9 groups (10 rats per group; 5 males and 5 females) based on the feed administered. One control group was fed with wheat flour-based feed, while another control group was fed with a supplemented diet containing FW and P (FW + P). The remaining four groups received different combinations of contaminated flours (AFB1, OTA), FW, and P. Each group, consisting of ten rats (five males and five females to evaluate sex differences), was fed for 28 days with the specific diet assigned to each group, with water available ad libitum. The diets were prepared in the laboratory and the full mycotoxin content was reported by Vila-Donat et al. [23]. Once the exposure time elapsed, Wistar rats were euthanized with isoflurane by inhalation, and their organs were stored at −80 °C.

The concentrations of AFB1 and OTA in the experimental feeds were determined based on the natural fungal contamination of barley and corn, respectively, by *Aspergillus flavus* and *Aspergillus steynii*. For feed preparation, barley and corn were naturally inoculated with *Aspergillus flavus* ITEM 8111 (from ISPA, Bari, Italy) and *Aspergillus steynii* 20510 (from CECT, Valencia, Spain) and cultured under optimal laboratory conditions to produce the respective mycotoxins. Mycotoxin concentrations in feed resulted from this natural contamination, simulating a realistic scenario of daily ingestion according to Mediterranean dietary patterns and human exposure estimates [24].

The concentrations of mycotoxins in the feed ranged around 4.5–4.9 µg/g for AFB1 and 5.4–8.8 µg/g for OTA, adjusted according to natural fungal growth and consistent with the targeted exposure doses. FW and P were incorporated as synergistic bioactive components to emulate functional food strategies typical of Mediterranean nutrition.

The actual exposure levels were calculated by considering the measured mycotoxin concentrations in the feed, the average daily feed intake, and the body weight of the rats during the fourth week of the trial. The target exposure levels were selected based on previously reported doses used in toxicity studies in rats, specifically 250 µg/kg bw/day for AFB1 [25] and 300–500 µg/kg bw/day for OTA [26]. These doses were chosen to simulate a realistic, sub-chronic exposure scenario, allowing the investigation of toxic and preventive effects without inducing acute toxicity. Body weight was measured weekly to monitor weight progression throughout the study.

### 2.3. RNA Extraction

To extract the RNA, approximately 50 mg of duodenum tissue was used for each sample. The samples were subsequently homogenized with Trizol™ (1 mL) and (0.2 mL) of chloroform using a digital homogenizer T10 Ultra-Turrax (IKA, Staufen, Germany). After centrifugation for 15 min at 4000 rpm and 4 °C, the extracted RNA was purified using the commercial ReliaPrep™ RNA Cell Miniprep Systems kit (Promega, Madison, WI, USA). The RNA was recovered in a final reaction volume of 30 µL with nuclease-free water. It was then evaluated via spectrophotometry using a NeoDot UV/VIS Nano Spectrophotometer by NeoBiotech (Nanterre, France Quimigen, Madrid, Spain), where it was quantified at 260 nm, and its quality was verified by the absorbance ratios at λ = 260/280 nm and λ = 260/230 nm. To ensure a common concentration for all samples, the concentrations were adjusted to 100 ng/µL with nuclease-free water. After evaluating the RNA spectrophotometrically, the concentrations were found to be between 155.1 and 4899.4 ng/µL, with appropriate 260/280 nm and 260/230 nm ratios both around 2, as shown in Table S1.

### 2.4. Reverse Transcription and qPCR Parameters

Each RNA sample was used to obtain cDNA following the instructions of the TaqMan Reverse Transcription Reagents kit (Thermo Fisher Scientific, Waltham, MA, USA). The reaction mixture underwent an initial heating step at 25 °C for 10 min. Reverse transcription was carried out at 37 °C for 30 min. Finally, enzyme inactivation was performed by heating at 95 °C for 5 min.

For the qPCR amplification, the reactions were performed using a QuantStudio™ 5 real-time PCR instrument (Thermo Fisher Scientific, Waltham, MA, USA) in 96-well plates with SYBR Green detection chemistry. Each reaction had a final volume of 10 µL, consisting of 5 µL of 2× Fast SYBR Green (Applied Biosystems, Waltham, MA, USA), 2 µL of primers (500 µM) (Invitrogen, Carlsbad, CA, USA), and 3 µL of cDNA. The gene S18 was used as the reference gene. The cycling conditions were set as follows: initial denaturation at 95 °C for 10 min to activate Taq DNA polymerase, followed by 40 cycles of denaturation at 95 °C for 15 s, annealing at 60 °C for 1 min for the genes p53, Hmox1, Occludin and NF-κB, and 58 °C for the gene Bax, with an extension at 72 °C for 15 s.

The standard curve for each gene was generated prior to the qPCR experiment under the same cycling conditions, using eight serial dilutions of cDNA. Amplifications were performed in triplicate for each dilution to assess the efficiency and linearity (R^2^) of the PCR reactions (Table 1).

### 2.5. Statyistical Analysis

The statistical analysis was performed by SPSS 24.0 (IBM Corp., Armonk, NY, USA). The median Log_2_RQ of all genes was calculated for each group, with control 1 considered as the reference group, having a Log_2_RQ value of 0. The normalized Cp was calculated per sample as ΔCt (experimental Ct − average housekeeping Ct) using the Ct values obtained from qPCR. A Student’s *t*-test was applied to assess the differences between the control groups and the samples that have been exposed to mycotoxins or mycotoxins associated with FW and P, considering *p* < 0.05 as statistically significant.

## 3. Results

### 3.1. Body Weight Gain and Duodenum Variations in Rats

**Figure 1:**
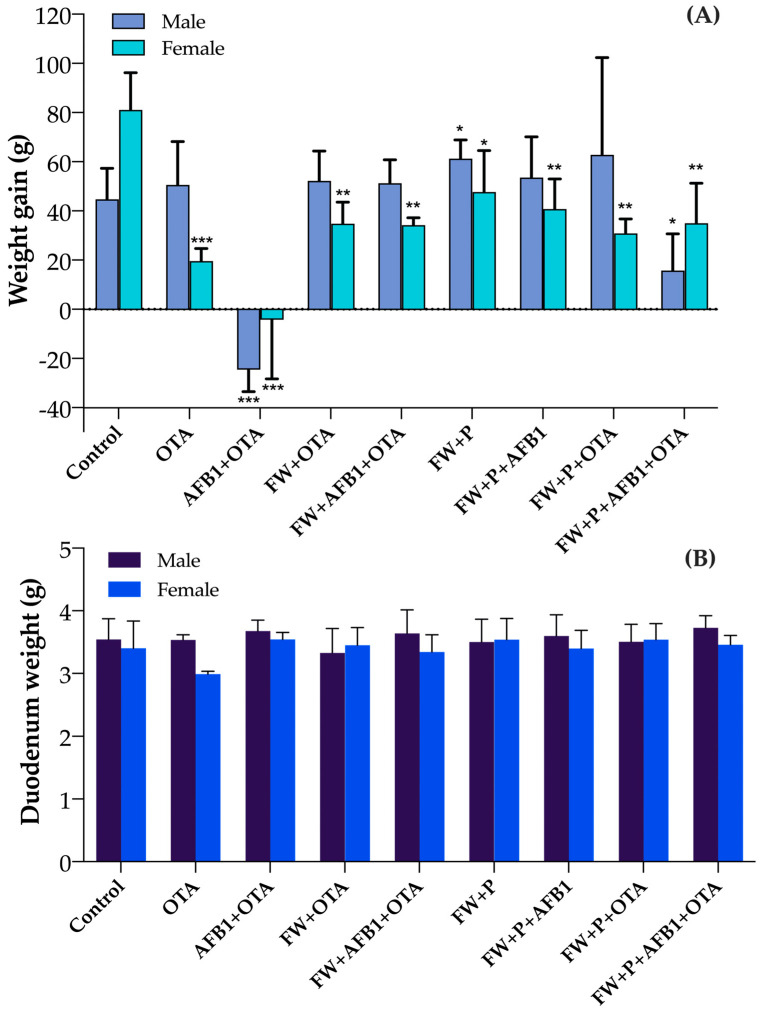
Body weight gain (g) from week 1 to week 4 (**A**) and duodenum weight (**B**) in male and female rats across experimental groups. Data reported as mean values among individuals ± SD. SD, standard deviation. * *p* < 0.05, ** *p* < 0.01, *** *p* < 0.001 statistically significant from the control.

Body weight gain was evaluated from week 1 to week 4 in both sexes across all experimental groups. In the control group, weight gain was higher in females (81.1 g ± 15.08) than in males (44.76 g ± 12.54). Exposure to OTA resulted in a moderate increase in weight for males (50.6 g ± 17.59), whereas females exhibited a slight increase in weight (19.6 g ± 5.13). The combined exposure to AFB1 and OTA (AFB1 + OTA) had the most pronounced toxic effect, causing a weight loss in males (−24.58 g ± 8.88) and a slight weight loss in females (−4.22 g ± 24.09). In contrast, the co-administration of FW with OTA or with the toxin mixture (FW + OTA and FW + AFB1 + OTA) notably improved weight outcomes in both sexes compared to the AFB1 + OTA group. The group receiving FW + P alone showed the highest gain in males (61.26 g ± 7.60), while females also benefited (47.74 g ± 16.78). The addition of FW + P to AFB1 exposure (FW + P + AFB1) resulted in improved weight gain in both sexes, indicating a partial protective effect. Interestingly, in the FW + P + OTA group, males exhibited the highest weight gain among all groups (62.85 g ± 39.47), while females showed moderate improvement (30.87 g ± 5.88). Finally, the group exposed to the full combination of mycotoxins and protective ingredients (FW + P + AFB1 + OTA) showed a positive effect compared to the AFB1 + OTA group, particularly in females (35 g ± 16.29), although male gain remained limited (15.8 g ± 14.85) (Figure 1A).

On the other hand, duodenal weight (Figure 1B) varied among experimental groups and between sexes. In the control group, males and females showed similar duodenal weights (3.54 ± 0.33 g and 3.40 ± 0.44 g, respectively). A clear reduction was observed in OTA-exposed females (2.99 ± 0.04 g), suggesting a higher sensitivity to this mycotoxin compared to males (3.54 ± 0.08 g). Interestingly, in the AFB1 + OTA group, both males and females exhibited relatively high duodenal weights (3.68 ± 0.18 g and 3.54 ± 0.11 g, respectively), possibly indicating a compensatory or inflammatory tissue response. In the FW and FW + P treated groups, duodenal weights were generally stable or improved. For example, in the FW + P + OTA group, females showed higher values (3.54 ± 0.25 g) than males (3.51 ± 0.28 g), and in the FW + P + AFB1 + OTA group, males reached the highest overall mean (3.73 ± 0.19 g), with females also showing recovery (3.46 ± 0.15 g). These findings support the hypothesis of a protective effect of FW and FW + P, particularly in females. Single values of individuals are reported in the supplementary material.

### 3.2. Differential Gene Expression of Apoptosis Key Genes Resulting from Exposure to OTA and FW

Exposure to OTA (Table 2) led to a marked upregulation of key regulatory genes in both males and females. In males, the genes Bax, NFKB, and Hmox-1 were significantly upregulated, highlighting a potential activation of pathways linked to apoptosis and oxidative stress. Similarly, in females, the genes p53, NFKB, and Hmox-1 showed substantial overexpression, suggesting comparable stress and damage responses. Interestingly, the addition of FW (FW + P) demonstrated a protective effect, effectively normalizing the expression of Bax in males and Hmox-1 in females to levels observed in the control group.

### 3.3. Differential Gene Expression of Apoptosis Key Genes Resulting from Exposure to AFB1 + OTA and FW

In male rats, the combination of AFB1 and OTA led to a downregulation of Bax, indicating an impairment of apoptosis pathway. In contrast, in female rats, the same exposure resulted in an overexpression of p53, suggesting a more pronounced activation of apoptosis. This highlights that in both males and females, mycotoxin exposure disrupts normal apoptotic processes when compared to the control group.

Upon the introduction of FW in the FW + AFB1 + OTA group, a reversal of effects was observed in both sexes. In males, FW led to an overexpression of all studied genes, including the previously downregulated Bax, alongside a significant overexpression of NF-κB, indicating a strong protective response. In females, FW caused a downregulation of p53 and Hmox1, while Bax was upregulated, suggesting a differential, yet protective, mechanism in mitigating the toxic effects of the mycotoxins (Table 3).

### 3.4. Differential Gene Expression of Apoptosis Key Genes Resulting from Exposure to FW + P

In male rats, the combination of FW + P led to a downregulation of p53 and an upregulation of NF-κB, suggesting a reduction in apoptosis compared to the control group. This indicates that these bioactive ingredients may enhance the stress response and reduce apoptotic activity. In the FW + P + AFB1 group, a further downregulation of p53 was observed, reinforcing the protective effect of these ingredients against AFB1 toxicity by limiting apoptosis. Conversely, in female rats, no significant changes were detected in either the FW + P or FW + P + AFB1 groups, indicating a less pronounced or negligible protective effect in females. This highlights a sex-specific response to the combination of FW and P (Table 4).

In male rats, the presence of OTA (FW + P + OTA) led to a marked downregulation of Bax, with no significant changes observed in p53, NF-κB, or Hmox-1 with the presence of AFB1 and OTA. This suggests that FW + P effectively mitigates the toxic effects of these mycotoxins, particularly in regulating apoptosis. Conversely, in female rats, the introduction of mycotoxins (FW + P + OTA) resulted in a significant downregulation of p53, indicating a disruption in the apoptotic pathway. Both Bax and Hmox-1 showed notable upregulation in the FW + P + OTA and FW + P + AFB1 + OTA groups compared to the control (FW + P), suggesting an enhanced stress response in females (Table 5).

### 3.5. Differential Gene Expression of Occludin Gene Resulting from Exposure to Mycotoxins and Bioactive Ingredients

**Figure 2:**
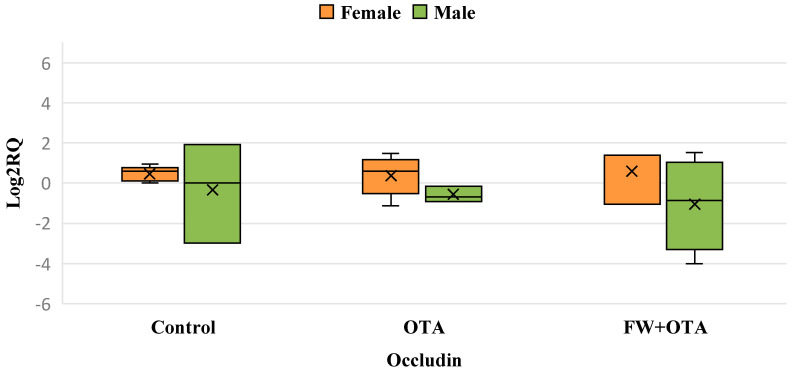
Box plot showing relative expression of Occludin in duodenum (male and female) when compared to control, to exposure with OTA, and OTA associated with FW (FW + OTA) by quantitative PCR (qPCR). RQ, relative quantification. Horizontal line inside each box represents the median value, while the X indicates the mean value of the data.

Exposure to OTA did not lead to any significant changes in occludin gene expression compared to the control group in both males and females. However, in males, a reduction in the biological variability of occludin expression was observed when compared to both the control and FW + OTA groups (Figure 2).

**Figure 3:**
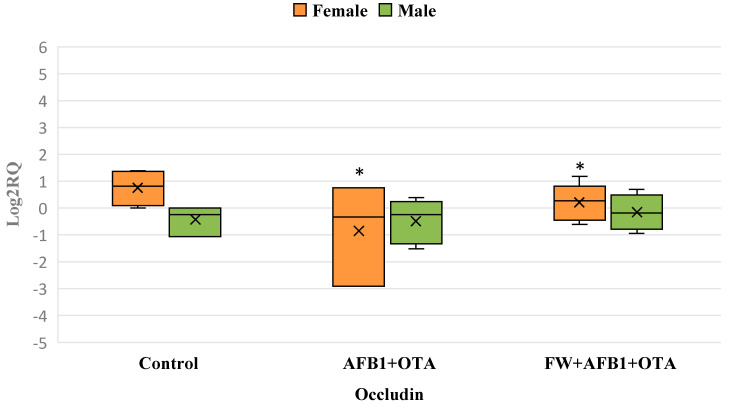
Box plot showing relative expression of Occludin in duodenum (male and female) when compared to control, to exposure with the mixture (AFB1 + OTA), and the mixture associated with FW (FW + AFB1 + OTA) by quantitative PCR (qPCR). RQ, relative quantification. Horizontal line inside each box represents the median value, while the X indicates the mean value of the data. * *p* < 0.05 significantly different from the control.

In the AFB1 + OTA group, occludin expression is significantly downregulated in females, with no significant changes observed in males. The addition of FW in the female group reduces variability, suggesting a stabilizing effect, but does not restore expression levels. No significant effects are observed in the male group across treatments (Figure 3).

**Figure 4:**
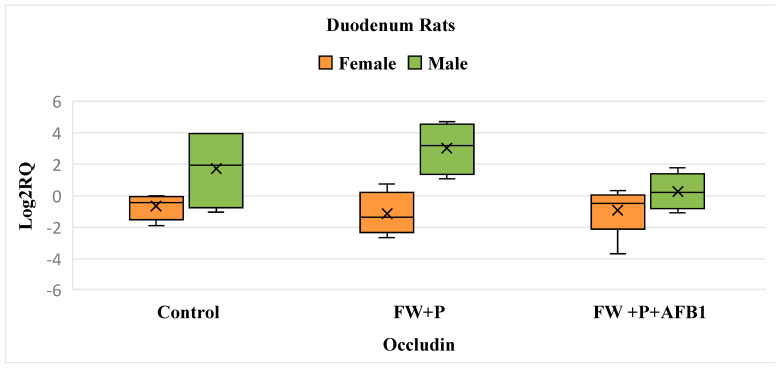
Box plot showing relative expression of Occludin gene in duodenum (male and female) when compared to control, to exposure with the mixture (FW + P), and the mixture associated with AFB1 (FW + P + AFB1) by quantitative PCR (qPCR). RQ, relative quantification. Horizontal line inside each box represents the median value, while the X indicates the mean value of the data.

In the FW + P group, there is no significant change in occludin expression compared to the control group for both sexes. Similarly, in the FW + P + AFB1 group, AFB1 exposure does not induce any notable effect, as occludin expression remains comparable to the control levels in both males and females. This suggests a potential mitigation of AFB1’s effects by the two bioactive ingredients (Figure 4).

**Figure 5:**
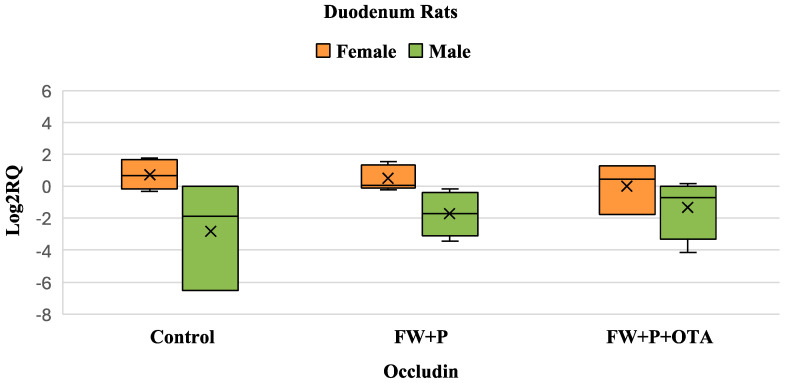
Box plot showing relative expression of Occludin gene in duodenum (male and female) when compared to control, to exposure with the mixture (FW + P), and the mixture associated with OTA (FW + P + OTA) by quantitative PCR (qPCR). RQ, relative quantification. Horizontal line inside each box represents the median value, while the X indicates the mean value of the data.

As observed in the FW + P + AFB1 group, no significant changes in occludin expression were detected in the FW + P + OTA group for both males and females (*p* > 0.05) (Figure 5).

**Figure 6:**
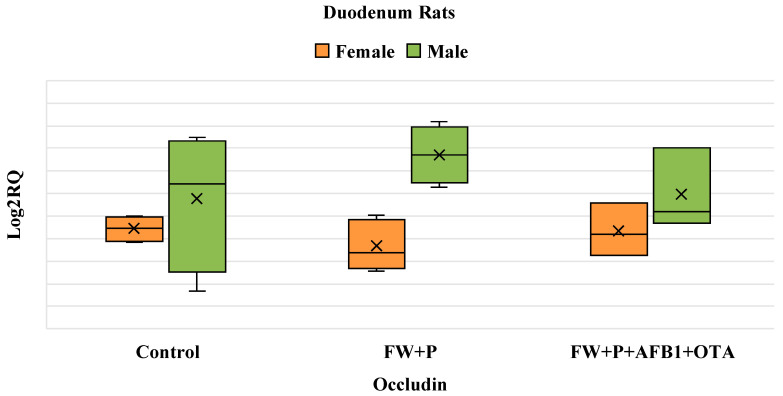
Box plot showing relative expression of occludin gene in duodenum (male and female) when compared to control, to exposure with the mixture (FW + P), and the mixture associated with AFB1 + OTA (FW + P + AFB1 + OTA) by quantitative PCR (qPCR). RQ, relative quantification. Horizontal line inside each box represents the median value, while the X indicates the mean value of the data.

In the FW + P + AFB1 + OTA group, there are no significant changes in occludin expression compared to the control for either males or females. This indicates that the combined exposure to FW + P and mycotoxins (AFB1 + OTA) does not significantly alter occludin expression in the duodenum for either sex (Figure 6).

## 4. Discussion

Research into the effects of AFB1 and OTA has revealed a complex array of genotoxic consequences, implicating multiple organs in their pathogenic mechanisms [27,28]. These mycotoxins have been extensively studied for their adverse effects on liver and kidney function, as well as the gastrointestinal tract [29]. Investigations have delved into the intricate molecular pathways underlying these effects [14,30,31]. These studies have highlighted the multifaceted nature of the gene response to mycotoxins, encompassing apoptosis, inflammation, and oxidative stress pathways. Moreover, contemporary researchers are also focusing on studying the preventive effects of bioactive compounds at a transcriptomic level [32].

The p53 gene is a crucial tumor suppressor gene that plays a central role in DNA repair and cellular apoptosis. Upon stimuli such as DNA damage, p53 induces the expression of genes that halt the cell cycle to allow repair or, in more severe cases, activate metabolic pathways leading to apoptosis [33]. One of these downstream targets is the Bax gene, a pro-apoptotic member of the Bcl-2 family. Bax promotes the release of pro-apoptotic molecules from the mitochondrial membrane, initiating the apoptotic cascade and ultimately causing cell death [34]. In the current study, OTA exposure resulted in the overexpression of p53 in females and Bax in males, demonstrating that OTA triggers apoptosis as a protective mechanism. This overexpression suggests that the body is activating a defense response to mitigate the cellular damage caused by OTA. Interestingly, simultaneous exposure to AFB1 and OTA led to a significant overexpression of p53 in females, further supporting the role of these toxins in inducing apoptosis.

Consistent with these findings, the overexpression of pro-apoptotic genes following mycotoxin exposure has been reported in numerous in vivo and in vitro studies. For instance, ref. [35] observed simultaneous exposure to AFB1 and deoxynivalenol (DON) upregulating pro-apoptotic genes, including p53 and Bax, in Caco-2 cells. Similarly, a study on rats revealed that exposure to Enniatins (ENs) led to an increased expression of p53 and Bax in the lower intestine [36]. These results reinforce the hypothesis that apoptosis serves as a cellular defense mechanism to eliminate damaged or potentially harmful cells [37].

Also, a significant downregulation of Bax was observed in males exposed to AFB1 + OTA. As Bax is pivotal for maintaining the balance between cell survival and cell death, this decrease suggests a harmful effect of the toxins, potentially disrupting the apoptotic process and favoring cell survival, which could lead to adverse outcomes.

NF-κB, beyond its anti-apoptotic role [38], is a pro-inflammatory transcription factor that plays a pivotal role in regulating cellular stress responses and inflammation [39]. Its overexpression in the duodenum of both male and female rats exposed to OTA suggests an activation of inflammatory pathways as part of the tissue’s response to the toxin-induced stress. This activation likely reflects the duodenum’s attempt to counteract OTA’s deleterious effects, including oxidative stress and damage to cellular components. A similar study conducted on porcine intestinal epithelial cells demonstrated that exposure to 4 µM OTA for 48 h significantly upregulated NF-κB expression, further reinforcing the link between OTA exposure and the activation of inflammatory signaling pathways [40].

Similarly to NF-κB, the Hmox-1 gene was significantly overexpressed in both male and female rats exposed to OTA. Hmox1 encodes heme oxygenase-1, a critical enzyme involved in antioxidant and anti-apoptotic activities that is upregulated in response to cellular stress. Its overexpression in this context can be attributed to the elevated production of reactive oxygen species (ROS) following OTA exposure, which is a hallmark of OTA-induced oxidative stress. This response aligns with its established role as a cellular defense mechanism aimed at counteracting oxidative damage and restoring redox balance [41].

In the current study, a slight but significant downregulation of the occludin gene was observed exclusively in female rats exposed to the combined toxins OTA + AFB1. Occludin, a critical gene of tight junctions that maintains the integrity and permeability of the intestinal epithelium [42], showed no significant changes in expression in males or in response to OTA alone in either sex. Despite the well-documented cytotoxic and pro-apoptotic effects of OTA and AFB1 on various cellular and molecular pathways, this finding indicates that the duodenal tissue shows resilience to these toxins with regard to occludin expression, except in the specific scenario of combined exposure in females.

The addition of FW alone to the groups exposed to mycotoxins demonstrated significant protective effects. In males, FW supplementation in the FW + OTA group restored Bax gene expression to levels comparable to the control group. Additionally, a marked overexpression of p53 was observed, indicating enhanced apoptotic activity, which is critical for controlling the proliferation of damaged or abnormal cells induced by OTA. Similarly, in males exposed to FW combined with AFB1 + OTA, Bax, initially downregulated by the combined mycotoxins, returned to control-like levels (no significant difference), accompanied by an overexpression of NF-κB.

In females, Hmox-1 expression was reduced in the FW + OTA group compared to OTA alone. The addition of FW restored its expression to normal levels (no significant difference compared to the control group). These findings highlight the importance of bioactive ingredients in mitigating the toxic effects of mycotoxins. Comparing gene expression between control and FW + P groups revealed slight sex-specific variations. In females, no significant differences were observed between the control group and FW + P. However, in males, p53 was significantly downregulated, while NF-κB was upregulated, suggesting reduced apoptosis in the duodenal cells of the exposed groups.

The preventive effects of these bioactive ingredients were particularly evident when combined with mycotoxins. In males exposed to AFB1 + OTA and FW + P (FW + P + AFB1 + OTA group), no significant differences in gene expression were observed compared to FW + P alone, underscoring the protective effects of the FW + P combination. Conversely, in females, a significant overexpression of Bax and Hmox-1 was observed in the FW + P + OTA and FW + P + AFB1 + OTA groups compared to FW + P, suggesting sex-specific differences in response. These results indicate that the FW + P combination was more effective in males than in females.

The two bioactive ingredients exhibited protective effects across all studied genes, except occludin, which was unaffected by the toxicity of AFB1 and OTA. These results are consistent with previous findings highlighting the potential of pumpkin and whey as functional ingredients. For instance, in a recent study evaluating bread matrices, both pumpkin and whey significantly reduced the intestinal bioaccessibility of AFB1 and OTA during in vitro digestion, with pumpkin achieving reductions up to 74% and 34%, respectively, and fermented whey showing reductions up to 68% for AFB1 and 20% for OTA. These reductions suggest that the bioactive compounds present in both ingredients may play a key role in mitigating the harmful effects of mycotoxins at the intestinal level, supporting the protective gene expression profiles observed in our in vivo model [43].

FW, known for its bioactive substances, including phenolic acids, peptides, and bacteriocins, has demonstrated antifungal, antimicrobial, and antioxidant properties [18]. Similarly, pumpkin contains antioxidants like carotenoids and vitamin E, which mitigate mycotoxin toxicity by scavenging free radicals and reducing oxidative stress. Additional components such as polysaccharides and sterols have also been implicated in reducing mycotoxin toxicity [44]. These bioactive properties have been validated in previous in vivo and in vitro studies, which demonstrated beneficial transcriptomic and molecular effects against AFB1 and OTA [45,46]. This study reinforces the potential of FW and pumpkin as preventive agents against mycotoxin-induced cellular and molecular damage.

Limitations of the current study include the lack of histopathological analysis and protein-level validation. These aspects will be addressed in future research to further strengthen the findings and provide a more complete picture of the protective effects of functional ingredients against mycotoxin-induced toxicity. This study represents a preliminary transcriptomic approach aimed at identifying early molecular changes in response to mycotoxin exposure and dietary supplementation. While mRNA expression data provide valuable insights into the activation of key pathways, we acknowledge the need for future studies to include histopathological and proteomic analyses for a more comprehensive assessment.

## 5. Conclusions

This study highlighted the significant preventive effects of the bioactive ingredients FW and Pumpkin on the expression of genes involved in apoptosis (p53 and Bax), inflammation (NF-κB), oxidative stress (Hmox-1), and tight junction integrity (occludin) in the duodenum in response to the mycotoxins AFB1 and OTA. FW and P supplementation restored the expression of genes disrupted by mycotoxins to levels close to those of the control groups, underscoring their protective role in mitigating the toxic effects of these compounds.

The results indicate that the preventive effects of FW and P are not sex-dependent, as the observed responses were generally comparable between males and females. However, two notable exceptions were identified. First, when comparing FW + P-exposed groups to controls, significant differences were observed in males for p53 and NF-κB expression, whereas no significant changes were noted in females. Second, while occludin expression was generally unaffected by AFB1 and OTA, a specific reduction was observed in females exposed to the combination of the two mycotoxins.

These findings highlight the preventive potential of FW and P in reducing the toxic impact of mycotoxins on key genes related to duodenal health, with generally similar protective effects across sexes. These results support the integration of FW and P into nutritional strategies to prevent mycotoxin-induced damage.

However, this study remains preliminary and is limited to transcriptomic analyses. To provide a more comprehensive understanding of the protective mechanisms activated by FW and P, further studies will be needed to include histopathological evaluations and protein expression analyses, such as Western blotting. These additional approaches will validate the gene expression results at the protein level and offer deeper insights into the molecular responses to mycotoxin exposure and bioactive ingredient supplementation.

## Figures and Tables

**Table 1:** Optimization parameters of the selected genes plus reference gene 18S rRNA.

Gene	NCBI Accession Number	Sequence	Annealing (°C)	Efficiency (%)	Linearity (R^2^)
18S rRNA	NM_213557.1	F: GAGCGTGTGATCACCATCAT R: TCCTTCACGTCCTTCTGTCT	60	111	0.993
p53	NM_030989.4	F: GTTCCGAGAGCTGAATGAGG R: TTTTATGGCGGGACGTAGAC	60	125	0.989
NF-κB	NM_017059.2	F: CTTCTCGGAGTCCCTCACTG R: CCAATAGCAGCTGGAAAAGC	60	108	0.981
Bax	NM_001276711.2	F: AAGAAGCTGAGCGAGTGTCT R: CAAAGATGGTCACTGTCTGC	58	104	0.991
Hmox1	NM_012580.2	F: GACGCATATACCCGCTACCT R: AAGGCGGTCTTAGCCTCTTC	60	99	0.990
Occludin	NM_031329.3	F: AGTACATGGCTGCTGCTGAG R: CCCACCATCCTCTTGATGTT	60	101	0.993

**Table 2:** Relative expression levels (log_2_RQFC ± SD) of apoptosis- and cell cycle-related genes in the duodenum of male and female rats after treatment with OTA and OTA combined with fermented whey (FW + OTA) compared to the control group, as measured by qPCR.

	Group	p53	Bax	NF-κB	Hmox-1
**Male**	Control	(−0.81 ± 2.07)	(−1.18 ± 2.04)	(0.30 ± 0.91)	(0.07 ± 0.61)
	OTA	(−0.04 ± 1.82)	(0.77 ± 1.52) *	(3.72 ± 0.52) **	(1.69 ± 1.25) **
	FW + OTA	(1.28 ± 2.12) *	(−0.95 ± 2.36)	(2.13 ± 1.49) **	(0.99 ± 0.84)
**Female**	Control	(−0.15 ± 0.73)	(−1.80 ± 1.57)	(1.82 ± 1.55)	(−0.19 ± 0.31)
	OTA	(4.62 ± 0.27) ***	(−2.34 ± 0.24)	(3.92 ± 0.99) ***	(2.06 ± 0.72) ***
	FW + OTA	(3.91 ± 0.88) ***	(−1.75 ± 0.71)	(3.61 ± 1.89) *	(0.80 ± 1.01)

RQ: relative quantification. * *p* < 0.05; ** *p* < 0.01; *** *p* < 0.001 significantly different from control (Student’s *t*-test).

**Table 3:** Relative expression levels (log_2_RQFC ± SD) of apoptosis- and cell cycle-related genes in the duodenum of male and female rats after treatment with AFB1 + OTA and the combined treatment with fermented whey (FW + AFB1 + OTA) compared to the control group, as measured by qPCR.

	Group	p53	Bax	NF-κB	Hmox-1
**Male**	Control	(−1.18 ± 2.13)	(−0.95 ± 2.58)	(−0.40 ± 1.92)	(1.68 ± 1.19)
	AFB1 + OTA	(−0.37 ± 1.26)	(−2.81 ± 2.23) *	(0.99 ± 1.63)	(1.19 ± 2.39)
	FW + AFB1 + OTA	(0.36 ± 2.07)	(−2.03 ± 2.63)	(2.19 ± 1.68) **	(2.01 ± 1.42)
**Female**	Control	(1.68 ± 1.30)	(−1.36 ± 1.76)	(0.70 ± 0.92)	(1.39 ± 1.38)
	AFB1 + OTA	(2.75 ± 1.46)	(−0.80 ± 0.83)	(0.70 ± 0.70)	(1.60 ± 1.01)
	FW + AFB1 + OTA	(1.73 ± 2.01)	(0.15 ± 2.19)	(0.96 ± 1.91)	(0.59 ± 1.44) *

RQ: relative quantification. * *p* < 0.05; ** *p* < 0.01, significantly different from control.

**Table 4:** Relative expression levels (log_2_RQFC ± SD) of apoptosis- and cell cycle-related genes in the duodenum of male and female rats after treatment with FW + P and FW + P + AFB1 compared to the control group, as measured by qPCR.

	Group	p53	Bax	NF-κB	Hmox-1
**Male**	Control	(−0.05 ± 0.37)	(−1.09 ± 1.37)	(−2.22 ± 1.92)	(1.15 ± 1.26)
	FW + P	(−2.14 ± 2.16) ***	(−0.76 ± 0.64)	(−0.53 ± 0.64) **	(1.64 ± 1.37)
	FW + P + AFB1	(−1.28 ± 1.99) ***	(−2.95 ± 1.52)	(−2.95 ± 1.47)	(0.57 ± 1.52)
**Female**	Control	(1.47 ± 1.19)	(−0.54 ± 0.47)	(1.27 ± 0.98)	(2.27 ± 2.09)
	FW + P	(1.87 ± 2.48)	(−0.23 ± 1.33)	(1.48 ± 2.12)	(0.97 ± 1.48)
	FW + P + AFB1	(0.81 ± 0.86)	(2.31 ± 2.30)	(2.31 ± 1.37)	(3.36 ± 1.51)

RQ: relative quantification. ** *p* < 0.01; *** *p* < 0.001, significantly different from control.

**Table 5:** Relative expression (Log_2_RQFC ± SD) of apoptosis and cell cycle-related genes in the duodenum of male and female rats following exposure to OTA (FW + P + OTA) and to the mixture of AFB1 + OTA (FW + P + AFB1 + OTA) compared to the FW + P group, as assessed by qPCR.

	Group	p53	Bax	NF-κB	Hmox-1
**Male**	FW + P	(−2.24 ± 1.89)	(−0.80 ± 0.84)	(−2.95 ± 2.86)	(−2.21 ± 1.53)
	FW + P + OTA	(−2.86 ± 2.19)	(−2.69 ± 0.62) **	(−3.24 ± 1.15)	(−2.90 ± 1.31)
	FW + P + AFB1 + OTA	(−1.76 ± 2.17)	(−1.21 ± 0.65)	(−2.55 ± 1.26)	(−2.29 ± 1.47)
**Female**	FW + P	(0.35 ± 0.79)	(1.56 ± 1.64)	(0.34 ± 0.90)	(0.64 ± 2.23)
	FW + P + OTA	(−0.52 ± 0.84) *	(2.76 ± 1.57) ***	(4.47 ± 1.46)	(5.15 ± 1.63) ***
	FW + P + AFB1 + OTA	(0.57 ± 0.94)	(4.16 ± 2.53) ***	(4.16 ± 2.53)	(3.92 ± 2.62) ***

RQ: relative quantification. * *p* < 0.05; ** *p* < 0.01; *** *p* < 0.001, significantly different from control.

## Data Availability

Not appliable.

## Supplementary Materials

The following supporting information can be downloaded at: https://www.mdpi.com/article/10.3390/foods14101793/s1, Table S1: RNA concentration and purity ratios for each sample. The table includes RNA concentration (ng/μl), and absorbance ratios 260/280 and 260/230, indicating protein and organic compound contamination, respectively. Samples are grouped by sex (male + female).

## Abbreviations

The following abbreviations are used in this manuscript:

AFB1	Aflatoxin B1;
AFBO	Aflatoxin B1 exo-8,9-epoxide;
Bax	BCL2-Associated X, Apoptosis Regulator;
Bcl-2	B-cell leukemia/lymphoma 2;
cDNA	Complementary DNA;
DNA	Deoxyribonucleic acid;
DON	Deoxynivalenol;
ENs	Enniatins;
FW	Fermented whey;
Hmox1	Heme oxygenase 1;
IARC	International Agency for Research on Cancer;
NF-κB	Nuclear factor kappa-light-chain-enhancer of activated B cells;
OTA	Ochratoxin A;
P	Pumpkin;
p53	Tumor protein p53;
RNA	Ribonucleic acid;
ROS	Reactive oxygen species;
RQ	Relative quantification;
RT-qPCR	Quantitative real-time PCR
